# Enhanced Amplification and Fan-Out Operation in an All-Magnetic Transistor

**DOI:** 10.1038/srep33360

**Published:** 2016-09-14

**Authors:** Saswati Barman, Susmita Saha, Sucheta Mondal, Dheeraj Kumar, Anjan Barman

**Affiliations:** 1 Department of Condensed Matter Physics and Material Sciences, S. N. Bose National Centre for Basic Sciences, Block JD, Sec. III, Salt Lake, Kolkata 700106, India

## Abstract

Development of all-magnetic transistor with favorable properties is an important step towards a new paradigm of all-magnetic computation. Recently, we showed such possibility in a Magnetic Vortex Transistor (MVT). Here, we demonstrate enhanced amplification in MVT achieved by introducing geometrical asymmetry in a three vortex sequence. The resulting asymmetry in core to core distance in the three vortex sequence led to enhanced amplification of the MVT output. A cascade of antivortices travelling in different trajectories including a nearly elliptical trajectory through the dynamic stray field is found to be responsible for this amplification. This asymmetric vortex transistor is further used for a successful fan-out operation, which gives large and nearly equal gains in two output branches. This large amplification in magnetic vortex gyration in magnetic vortex transistor is proposed to be maintained for a network of vortex transistor. The above observations promote the magnetic vortex transistors to be used in complex circuits and logic operations.

In magnetic thin films, magnetostatic interactions usually force the magnetization to lie parallel to the plane of the film. For laterally confined thin ferromagnetic platelets satisfying certain conditions of geometric and magnetic parameters, the magnetic moments form rotationally symmetric patterns that follow an in-plane flux closure configuration known as magnetic vortex. Due to the closed flux lines, the dipolar energy is minimized and the structure is stable. However, at the centre of the disk, magnetization cannot remain flat, because the short-range exchange interaction favours a parallel alignment of neighbouring magnetic moments. The exchange energy forces the magnetization to stay out-of-plane in a very small area of only a few exchange lengths in diameter and thus creates the vortex core with a distinct polarization *p*, either up (*p* = +1) or down (*p* = −1)^1^,^2^,^3^. Magnetic vortices can be brought to gyration with a frequency in the sub-GHz to GHz range by the application of the magnetic fields and spin polarized currents^4^,^5^,^6^,^7^,^8^,^9^,^10^,^11^,^12^,^13^,^14^,^15^,^16^,^17^. The sense of gyration (clockwise or counter-clockwise) is determined by the vortex core polarization. Recently, dynamic vortex core reversal was also obtained by application of pulsed magnetic fields^18^,^19^,^20^,^21^,^22^, alternating magnetic fields^14^ or spin-polarized currents^23^,^24^,^25^,^26^,^27^,^28^,^29^,^30^. These findings opened up the possibility of using magnetic vortices as memory bits and for future spintronic applications. Wide investigations of the physics behind this reversal mechanism have also been started.

The vortex core gyration is qualitatively described by the Landau-Lifshitz-Gilbert equation. In the linear regime, the vortex core equation of motion can be obtained from Thiele’s equation^31^. During the motion of the vortex core, the demagnetization field at the core points antiparallel to the enlarged domain generated due to the displaced core and the time derivative of magnetization vector at the core points into the disk centre or into the opposite direction. A variation in chirality naturally changes the direction of the demagnetization field and therefore it does not affect the core gyration direction. Therefore, the core gyration direction is solely controlled by the core polarization. Since the vortex core motion is governed by the demagnetization field, the demagnetization energy determines the core potential. In the linear regime, parabolic potential can be assumed and core can be described under a harmonic oscillator model^32^. Therefore, magnetostatically coupled vortex gyration in the linear regime can be considered as the coupled forced oscillator. Consequently, one expects mutual energy transfer between the gyrating vortices in the linear regime and a constant phase relation exists between the gyrating vortices^33^,^34^.

The mutual transfer of energy between uniformly excited magnetostatically coupled vortices due to the gyrotropic motion is extremely important in devices for microwave communication^35^. Dynamical property of coupled vortices in two nanodisks has been investigated analytically, numerically and experimentally^4^,^10^,^33^,^34^,^35^,^36^,^37^,^38^,^39^. The magnetostatic interaction between two closely spaced vortices is found to play an important role in determining the dynamics of vortices. The rigid vortex model has been employed to calculate the magnetostatic interaction between the coupled vortices^4^. The eigenfrequencies of circular vortex core motion are found to depend on polarizations of the cores. The chiralities of the vortices do not influence the eigenfrequencies. It is found that the time-averaged magnetostatic interaction energy varies as the minus sixth power of the separating distance. Therefore, the dynamical system of coupled vortices can be compared with a diatomic molecule with the van der Waals bonding induced by the dipolar interaction. Magnetic charges on the side surfaces of the off-centred nearby vortices induce the magnetic dipole-dipole interaction. The splitting of gyration mode of coupled vortex system into higher and lower frequency modes via dipolar interaction has been demonstrated experimentally^36^. The main mode splitting is due to a chirality sensitive phase difference in gyrations of the coupled vortices, whereas the magnitude of the splitting is determined by their polarity configuration. It has been shown that the coupled pair of vortices behaves similar to a diatomic molecule with bonding and antibonding states. Selective excitation of coupled gyration modes has been performed by spin transfer torque^35^. For coupled gyration, the asymmetric variation in resonance frequency with respect to the external magnetic field for same polarization and opposite chirality implies a transition from stronger to weaker magnetostatic coupling. In contrast, for same polarization and chirality, the symmetric variation in resonance frequency implies that the strength of the magnetostatic interaction is not modified. Locally excited magnetic vortex gyration propagation through a linear array of magnetostatically coupled nanomagnetic discs is mediated by the magnetic side charges and can be controlled by combination of polarization, chirality and shape^33^,^34^. Transmitted peak amplitude and velocity of propagation is maximum for identical core polarization and chirality of the nanodiscs with geometric asymmetry.

Experimental demonstration of all the collective normal modes^10^ and logic operations^38^ were recently achieved. Analytical and numerical calculations indicate that the energy transfer rate is characterized by the relative polarization configuration, saturation magnetization, aspect ratio of the magnetic disks and core to core distance^37^ and attenuation is governed by the intrinsic damping constant and other physical parameters.

The signal transfer is found to be maximum when the core polarizations of the vortices are opposite^10^ but none of the above works showed an amplification in the output signal, which is important for signal propagation over longer distance through complicated circuitry. If the input amplitude is made high to overcome this problem, the vortex motion enters into nonlinear regime and vortex core switching occurs, making it difficult to increase and maintain a larger amplitude output and a constant phase relation between dynamics of input and output vortices. On the other hand, if the input vortex is resonantly excited with small amplitude signal, the amplitude of the response gradually increases indicating that the core switching will occur at some point, which is also not desirable for device applications. One alternative is to use a very small amplitude off-resonant signal (within 5% of the resonance frequency) to get around this problem^35^.

However, a recent work by Kumar *et al*. shows an amplification in the transferred signal in magnetostatically coupled vortices, paving the way towards a magnetic vortex transistor (MVT)^39^. Antivortex soliton dynamics moving through the inter-disk stray field is proposed to be responsible for this gain of transferred energy. A three vortex sequence was proposed for the MVT where the left vortex (emitter) is locally excited and the output gain on the extreme right vortex (collector) can be switched from an ON to OFF state by controlling the core polarity of the middle vortex (base) in analogy to an electronic bipolar junction transistor^39^. However, the maximum gain obtained in ref. 39 is about 15 dB and an attempt to show fan-out operation remained unsuccessful. With the miniaturization of devices and complexity of the circuits it is imperative to obtain larger gain and fan-out operation. The gain, which is defined as the difference between the energy spectral densities (ESD) at the output and input, is expected to depend upon a number of parameters such as the distance between the three vortices, excitation frequency, aspect ratio of the magnetic disks and symmetry of the arrangement of three vortices. On the other hand, the failure towards fan-out operation was because the antivortex solitons do not split easily to transfer energy equally to both symmetrically placed branches. Creation of topological instability in stray field antivortices by introducing positional asymmetry may forbid any preferential energy transfer leading to successful fan-out operation.

Here, we show a large increase in the gain of the MVT by tailoring the inter-disk separation and the arrangement asymmetry. The absorption of anivortex solitons at the neighbouring vortex and absence of a feedback loop is found to be responsible for this high gain. Furthermore, the output from this asymmetric magnetic vortex transistor (AMVT) is fed into the input of another two such AMVTs to perform a successful fan-out operation and significantly large and nearly equal gains are observed at both the outputs.

## Results

**Figure 1:**
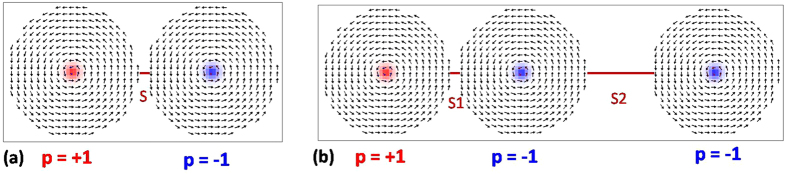
The ground state spin configuration of magnetic disk of diameter of 200 nm and thickness of 40 nm arranged in coupled (**a**) two vortex system with inter disk separation (*S*) 10 nm and (**b**) three vortex system with the separation between left and centre vortex (*S*1) of 10 nm and the separation between centre and right vortex (*S*2) of 100 nm.

A circular disk of 200 nm diameter and 40 nm thickness made up of Ni_80_Fe_20_ (Py) is used to form a stable magnetic vortex. The coupled two vortex system is formed by placing another Py disk of same dimension next to the first disk and similarly three vortex system is formed by placing three such disks in a row. Figure 1(a,b) show coupled two and three vortex systems with core polarities (+1, −1) and (+1, −1, −1), respectively. Spatially averaged x-component of magnetization 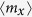 is considered as an indicator of core displacement away from its equilibrium position. The square of the amplitude of Fourier transform of 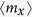 is considered as the energy spectral density (ESD), which shows the peak power corresponding to maximum core displacement.

### Coupled Vortex Pair

**Figure 2:**
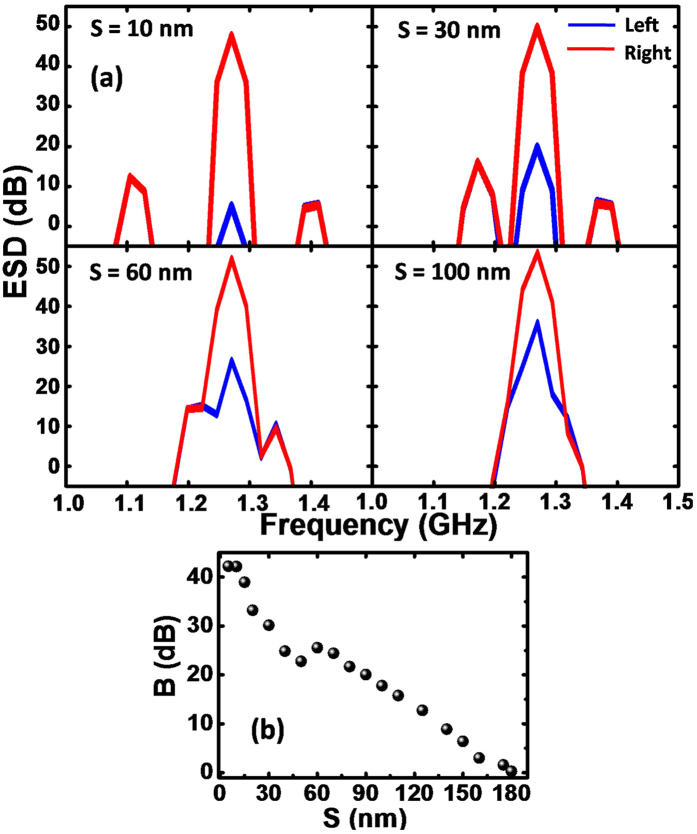
(**a**) Energy spectral densities (ESDs) for coupled two vortex systems with varying inter-disk separation (*S*). (**b**) Variation of gain (*B*) with *S* for coupled vortex.

Before optimizing the MVT gain and other related MVT operations, we investigated the energy transfer gain in a coupled two vortex system as a function of inter-disk separation. Figure 2(a) shows the ESDs for the left and right vortex for the coupled two vortex systems with four different inter-disk separations. Here, only the left vortex is excited with a local field. For higher separations (60 and 100 nm) a single gyration mode is observed, while for lower separation three coupled modes are observed. The mode splitting is an interesting problem and is studied in details in previous literature^11^,^12^,^13^,^35^,^36^. However, here only the central peak corresponding to highest ESD is considered for calculating the energy transfer gain. The gain is calculated as the difference between maximum value of ESD of right and left vortex. For all four separations large gain is observed. The gain (*B*) is systematically plotted as a function of inter-disk separation (*S*) in Fig. 2(b). Here, *B* shows almost a linear increase with the decrease in *S* and reaches 25.55 dB at *S* = 60 nm. For *S* < 60 nm, *B* suddenly decreases to 22.77 dB, followed by another steeper increase down to *S* = 10 nm, where it reaches a maximum saturation value of 42.15 dB and does not increase any further. In order to justify the choice of core polarities, we changed the core polarities of the coupled vortex system with separations of *S* = 10 nm and 60 nm to (+1, +1) and observed a much reduced gain in the latter cases (22.0 dB for *S* = 10 nm and 7.0 dB for *S* = 60 nm) as opposed to 42.15 dB (*S* = 10 nm) and 22.77 dB (*S* = 60 nm) for the former cases. Greater details of this observation can be found in the supplementary Figure 1S(a).

**Figure 3:**
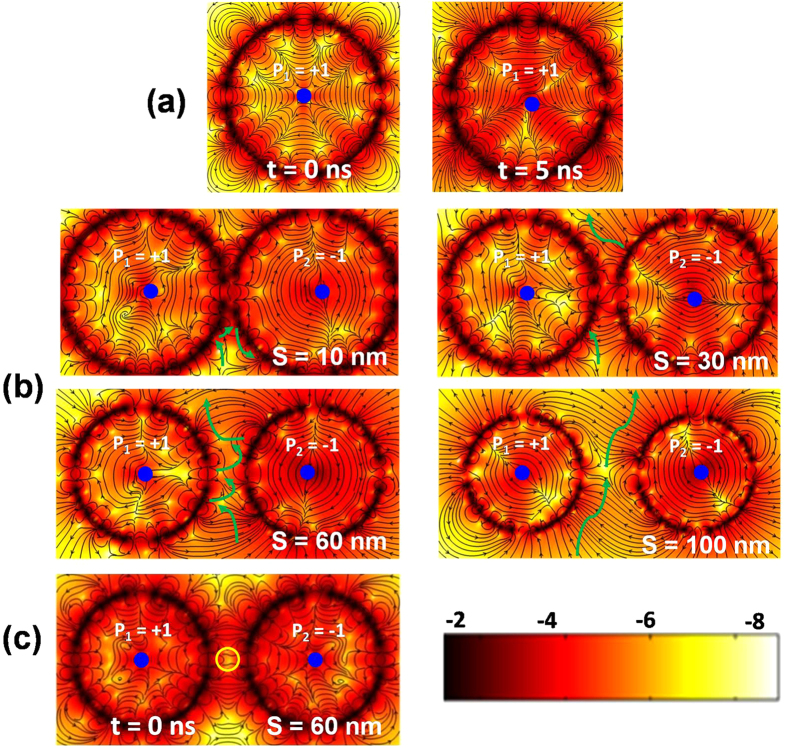
Stray field distributions for (**a**) single vortex at time t = 0 and 5 ns and (**b**) magnetostatically coupled two vortex systems with four different inter-disk separations are shown. The green arrows represent the paths of the antivortex solitons. (**c**) The ground state strayfield configuration of a magnetostatically coupled two vortex system with inter-disk separation 60 nm at t = 0 ns is shown and the antivortex is marked by a yellow circle. The colour bars are shown at the bottom of the figure. The contour colouring is based on the sum of squares of x and y components of the stray field and the colour bar is in dB. The core of the vortex is marked by blue dot.

To understand this non-monotonic increase in *B* with *S* we have studied the temporal evolution of the stray magnetic field. Figure 3(a) shows the stray field distribution of a single vortex at two different times (t = 0 and 5 ns) and it is clear that the stray field distribution changes with time as the core of the vortex starts to gyrate even for a single vortex. Figure 3(b) shows the snapshots of the simulated stray fields for four different two vortex systems at specific instants of time of the dynamics. The supplementary movies M1–M4 show the dynamics in detail. Earlier work showed that the energy transfer from left to right vortex is mediated by stray field antivortices, which travels from the left disk and collides with the right disk. It is also inferred in ref. 39 from the only case studied *i.e., S* = 50 nm, that an amplification occurs when no feedback loop of stray field antivortex trajectory is present between the two disks. A feedback causes an energy rebalancing and hinders the amplification. However, a detailed study of variation of gain as a function of inter-disk separation, as presented here, shows that amplification may occur even in the presence of a feedback loop and even when the antivortex do not directly collide with the right vortex. For *S* = 100 nm, the antivortex generates after the onset of the dynamics and passes through the gap between the two disks without a single collision with either of the disks and the energy is transferred only due to the variation of the flux lines with time, which leads to a gain of 17.80 dB. With the reduction of *S,* collision of antivortex starts to occur with both disks, leading to a gain in the energy transfer albeit with a feedback, which hinders a very large value of the gain. The feedback mechanism weakens with further reduction in *S*, causing a continuous increase in gain value. However, for *S* close to 60 nm a special situation occurs where a stray field antivortex is present even in the ground state before the onset of the dynamics, as shown by the yellow circle in Fig. 3(c), and the energy transfer occurs immediately after the onset of the dynamics causing the feedback mechanism to weaken further. However, when *S* deviates significantly from 60 nm this antivortex disappears from the ground state and the transfer of energy starts after the creation of a stray field antivortex following the onset of gyration in the left vortex. As *S* reduces further, the feedback mechanism and the ensuing energy rebalancing disappears and a one-way transfer of energy from the left to right vortex causes energy build up in the right vortex and an enhanced amplification occurs. This continues to increase with further reduction of *S* due to the faster transfer of energy. Finally a saturation occurs at *S* ≤ 10 nm, when a very high dipolar interaction energy between the nearest edge regions of the disks forbids the antivortex to enter that zone and it transfers energy only in the lower half of the disk.

### Magnetic Vortex Transistor (MVT)

**Figure 4:**
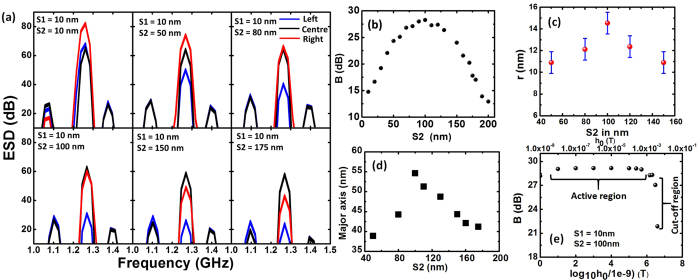
(**a**) ESDs for MVT and AMVT. (**b**) Variation of gain (*B*) with *S2* for AMVT. (**c**) Variation of relative core displacement (*r*) of the right vortex with respect to the left vortex with *S2* for AMVT. (**d**) Variation of length of the major axis of the ellipse with *S2* for AMVT. (**e**) Variation of gain (*B*) with strength of the applied magnetic field.

**Figure 5:**
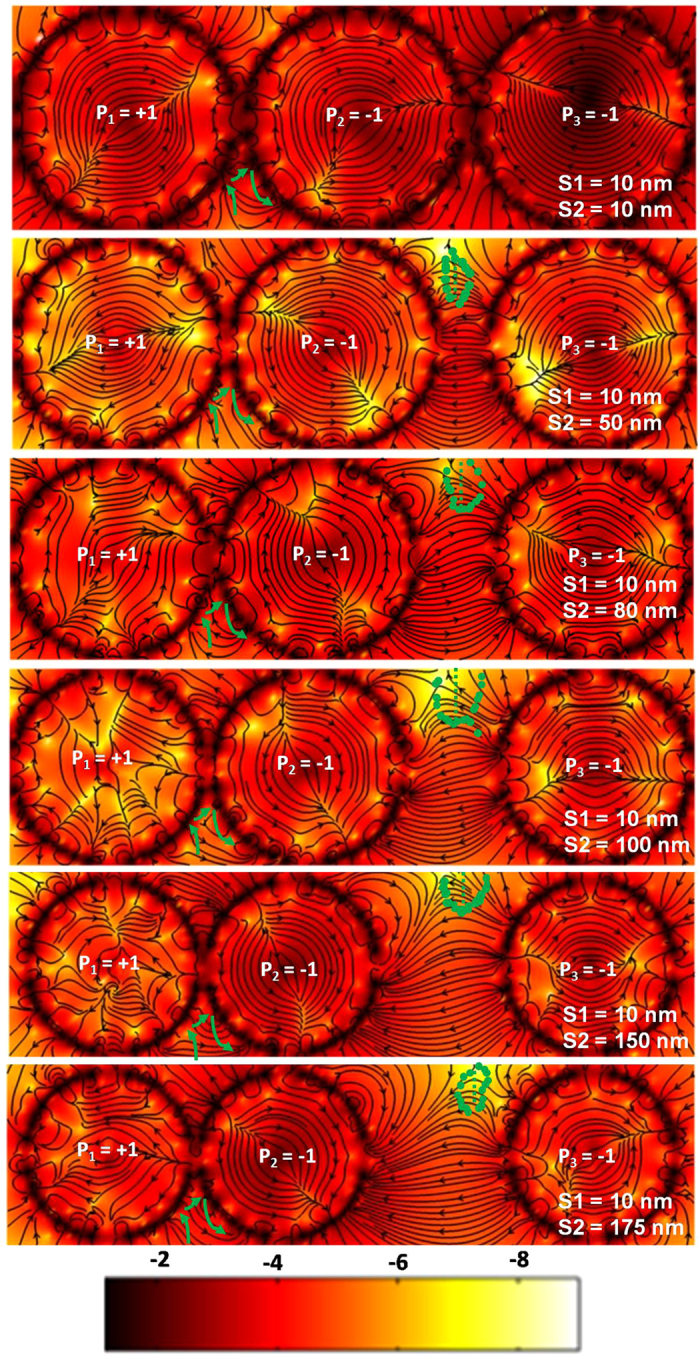
Stray field profiles for MVT and AMVT. The green lines represent the paths of the antivortex solitons. The colour bars are shown at the bottom of the figure. The contour colouring of is based on the sum of squares of x and y components of the stray field and the colour bar is in dB.

For the MVT operation, we begin with a three vortex sequence with polarizations +1, −1 and −1, which worked successfully in our previous work^39^. Here, we vary the distance between the left and middle vortex (*S1*) and the middle and right vortex (*S2*) to optimize the gain *B*. In symmetric MVT, maximum gain achieved was 14.8 dB for *S1* = *S2* = 50 nm^39^. Based on simulations by systematic variation of the values of *S1* and *S2* we show here that introduction of asymmetry in the arrangements of the three vortices lead to an enhanced gain in MVT and the gain can be maintained in complex circuits along different branches. The asymmetry is introduced by making the separation between the central vortex and right vortex (*S2*) larger than that between the left vortex and middle vortex (*S1*). The optimum value is obtained by fixing *S1* = 10 nm, which provides maximum gain for a coupled two vortex system. Various aspects of dynamics of MVT have been presented in Figs 4 and 5. Figure 4(a) shows the ESDs of MVTs for *S1* = 10 nm and for *S2* varying between 10 and 175 nm. Figure 4(b) shows that the gain increases with the increase in *S2*, and a maximum of 28.28 dB is observed at *S2* = 100 nm, beyond which it decreases with further increase of the *S2*. This two-fold increase in the gain in an AMVT is remarkable and can lead to applications in magnetoelectronic circuits requiring signal amplification. To justify the choice of core polarities we changed the core polarities of the three vortex system with separations of *S1* = 10 nm and *S2* = 100 nm to (+1, +1, +1), (+1, +1, −1) and (+1, −1, +1). We observed much reduced gain in these combinations of core polarities as opposed to the former case (+1, −1, −1). The gain in output vortex is −4.5 dB, 5.0 dB and 16.5 dB for the polarity combination (+1, +1, +1), (+1, +1, −1) and (+1, −1, +1), respectively. Greater details of this observation can be found in the supplementary Figure 1S(b). In order to understand the relation between the MVT gain with the relative core displacement (*r* = displacement of vortex core in disk 3 – displacement of vortex core in disk 1) we plotted, in Fig. 4(c), *r* as a function of *S2* for some selected values of *S2*. A similar nature in the variation of the relative core displacement with *S2* is observed to the variation of gain *B*. To understand this enhanced amplification we again investigated the temporal evolution of the magnetic stray field as presented in Fig. 5 while greater details of the dynamics can be found in the supplementary movies M5–M10. We observe that the mechanism of transfer of energy from the left to the center vortex for all cases is same as the coupled two vortex system with *S1* = 10 nm. However, the transfer mechanism from the center to the right vortex varies with *S2*. Consequently, the final gain of the vortex transistor is dominated by the stray field dynamics between the center and the right vortex. At *S1* = *S2* = 10 nm, the antivortices created between the center and the right vortex are very small in size and therefore, very little energy is transferred to the right vortex leading to a smaller gain of 14.76 dB. As *S2* increases, larger antivortices are created and they traverse nearly elliptical paths through the stray field between the center and the right vortex. The length of the major axis of this elliptical path appears to determine the amount of transfer of energy and it is plotted as a function of *S2* in Fig. 4(d). It is clear that this length increases with the increase in *S2* and reaches a maximum at *S2* = 100 nm beyond which it drops. This variation is consistent with the variation of gain and hence we believe they are strongly correlated. We further investigated the variation of gain of the optimized AMVT structure (*S1* = 10 nm and *S2* = 100 nm) with the input signal amplitude *h*_*0*_. Figure 4(e) shows a plot of variation of gain with logarithm of signal amplitude *h*_*0*_. Here, the core of the left vortex reversed for *h*_*0*_ = 5 mT and therefore, we limit ourselves to 4 mT. For lower values of *h*_*0*_, the gain remains nearly constant at *B*_*active*_ = 28.28 dB. This is considered as the active region of the AMVT and *B*_*active*_ resembles the small-signal gain of an electronic bipolar junction transistor. At 5 mT, the core of left vortex switches, and the gain suddenly drops, defining the cut-off region. Unlike the symmetric MVT the non-linear distortion of gain from *B*_*active*_at higher signal strength (before cut-off) is not observed in the AMVT.

### Fan-Out

**Figure 6:**
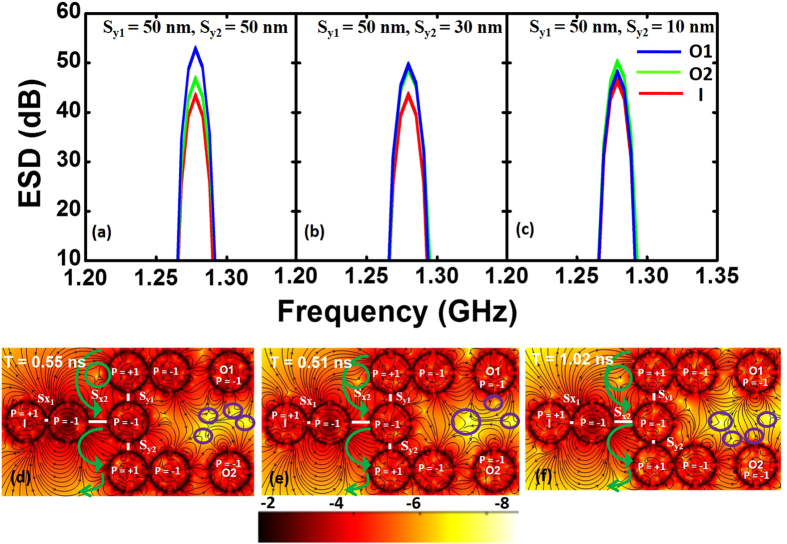
(**a–c**) ESDs for fan-out operation. (**d–f**) Stray field profiles for fan-out. The green lines represent the paths of the antivortex solitons and the antivortices are marked by green and violet circles. The interaction time (T) is mentioned in each figure. The colour bar is shown at the bottom of the figure. The contour colouring is based on the sum of squares of x and y components of the stray field and the colour bar is in dB.

The symmetric MVT did not show a successful fan-out operation as the antivortex soliton does not split easily leading towards an asymmetry in energy transfer in the two arms of the fan-out circuit. Here, we investigate the possibility of fan-out using AMVT. The optimized AMVT with the horizontal distance between the left and central vortex (*S*_*x1*_) as 10 nm and that between the central and right vortex (*S*_*x2*_) as 100 nm with similar core polarity (+1, −1, −1) like AMVT is used as a unit here. We placed two AMVTs vertically above and below the original AMVT while keeping the left vortex of the upper and lower branches and right vortex of the original AMVT in the same line and vary the vertical distances of the upper (*S*_*y1*_) and lower (*S*_*y2*_) AMVTs with the input AMVT to tune the gains at the two outputs. The ESDs and stray field profiles for fan-out circuits with different values of *S*_*y1*_ and *S*_*y2*_ are shown in Fig. 6. The detailed temporal evolutions of the magnetic stray fields are provided in supplementary movies M11–M13.

At first we started with a symmetric fan-out circuit by setting *S*_*y1*_ = *S*_*y2*_ = 50 nm. Gain is observed in both the outputs (O1 and O2) but gain in the upper branch is much higher than the gain in the lower branch (*B*_*O1*_ − *B*_*O2*_ = 6.33 dB). To remove this asymmetry in gain we have introduced a second level of asymmetry in the fan-out circuit. A systematic variation of *S*_*y1*_ and *S*_*y2*_ shows that for *S*_*y1*_ = 50 nm and *S*_*y2*_ = 30 nm the ESDs at O1 and O2 are nearly same and subsequently the gains are also the same, *B*_*O1*_ = *B*_*O2*_ = 5.4 dB. When *S*_*y2*_ is further decreased, the gain in O2 becomes higher than O1. Figure 6(c) shows that *B*_*O2*_ − *B*_*O1*_ = 2.77 dB.

To understand this behaviour, we have studied the stray field dynamics (Fig. 6(d–f)) of the fan-out circuits in details. An antivortex soliton is observed to be moving from the left vortex of the upper AMVT to the left vortex of the lower AMVT via the right vortex of the input AMVT. The size and speed of this antivortex soliton and interaction time (T) with right vortex of the input AMVT is determined by the amount of asymmetry in the structure. For the first case shown here (*S*_*y1*_ = *S*_*y2*_ = 50 nm), four antivortices marked by violet circles are observed close to the middle and right vortex of the upper and lower AMVTs (Fig. 6(d)). Among these four antivortices, two are situated between the right vortex of the upper and lower AMVTs. The larger antivortex is stationary at the halfway between the right vortex of the upper and lower AMVT branches and the smaller one moves towards the right vortex of the upper AMVT branch. As a result, the upper AMVT (O2) shows higher gain for symmetric fan-out circuit.

In the case of successful fan-out (*S*_*y1*_ = 50 nm, *S*_*y2*_ = 30 nm), the energy transfer mechanism is different from that in the symmetric case. Here we observe three antivortices marked by violet circles close to the middle and right vortices of the upper and lower branches (Fig. 6(e)). Two of them first combine and then again split into two antivortices. These two antivortices move towards the right vortex of upper and lower AMVT branches and transfer energy equally. In the third case (*S*_*y1*_ = 50 nm, *S*_*y2*_ = 10 nm), in addition to the above mentioned antivortices, there are very small antivortices near the right vortex of the lower branch (marked by violet circles), causing the energy transfer to be significantly higher in the lower branch as compared to the previous two cases (Fig. 6(f)).

In addition to the distribution of energy to the two outputs in a fan-out circuit, we also observe an interesting trend in the sum of energy transferred to the two outputs and its relation to the stray field antivortex dynamics. When *S*_*y1*_ = *S*_*y2*_, the antivortex soliton marked by green circle is quite large and it moves very quickly between the two branches (interaction time, T ≈ 0.55 ns). As a result, the sum of the output gains is large (O1 + O2 = 12.71 dB). As we break the symmetry by lifting up the lower branch slightly towards the input AMVT, the sum of the gains decreases to 11.67 dB. In this case, the size of the antivortex soliton (marked by green circle) moving from the left vortex of the upper AMVT branch to left vortex of the lower AMVT branch via the right vortex of the input AMVT decreases slightly as compared to that of the symmetric fan-out circuit. The interaction time (T) of about 0.51 ns is nearly same as that for the symmetric case and the sum of gain is also similar. As we increase the asymmetry further (*S*_*y2*_ = 10 nm), the size of the antivortex decreases (marked by green circle) further and it moves very slowly between the left vortex of the upper and lower branches of the circuit via the right vortex of input AMVT (interaction time, T ≈ 1.02 ns). As a result the sum of the gains at two output branches decreases significantly (O1 + O2 = 6.31 dB).

## Discussion

We have numerically explored the dependence of signal amplification of vortex core gyration in coupled vortex pair on the inter-disk separation with the goal of increasing the gain of a magnetic vortex transistor (MVT). We observe a non-monotonic increase in the amplification in the energy transfer with separation in the two-vortex case. An almost linear increase in the amplification occurs with the decrease in separation from higher separation down to 60 nm where the amplification suddenly drops followed by another steeper increase with further decrease in separation. The amplification saturates at a large value of 42.15 dB for the inter-disk separation of 10 nm. This amplification of energy transfer is extended to MVT consisting of a three vortex sequence with a particular combination of polarity of the three vortices (+1, −1, −1). We found that a symmetric arrangement of the three vortex sequence does not give the maximum gain of the transistor but an asymmetric arrangement is necessary. In this case, we found that a particular combination of separation i.e., 10 nm between the left and middle disk and 100 nm between the middle and the right disk gives the maximum gain of 28.28 dB, which is nearly a factor of two improvement as compared to previously reported value. This remarkable observation can be interpreted in terms of the temporal evolution of stray magnetic field and anivortex packets moving through the stray field is held responsible for the above observation. We postulated some mechanisms based upon the motion of antivortex solitons and this can successfully explain our numerical results in great detail.

One important issue of this study is the scalability of the magnetic vortex transistor. Numerical test studies by varying the diameter and thickness of the individual disks and the inter-disk separation could not produce any conclusive results and we believe a very thorough numerical study by varying each dimension would be necessary to establish the scalability. Development of analytical tools to calculate the magnetic vortex transistors would be helpful in drastically reducing the calculation time and in obtaining a conclusive result but this is beyond the scope of our study at present. Another important issue is the transistor operation timescales. Numerical analyses show that the gain of the optimized AMVT reaches a reasonably high value after about 10 ns and nearly saturates at about 20 ns from the excitation and does not fall within 40 ns of the simulation time. On the other hand from the literature^14^,^18^,^19^,^20^,^21^,^22^,^23^,^24^,^25^,^26^,^27^,^28^,^29^,^30^, we understand that vortex core reversal can occur within about 1 ns by applying current or Oersted field. This determines the timescale of switching the transistor from ON to OFF state and vice versa.

Further, we have numerically explored the fan-out operations using the asymmetric magnetic vortex transistor (AMVT). Total gain and distribution of gain among the two branches were observed to be dependent upon the vertical separation between the input AMVT and the branches. The mechanisms of movement of antivortex solitons moving through the dynamic stray field were explored. With the rapid advancement of nanoelectronic devices we hope that our findings will promote the design of complex circuits entirely based on magnetic vortices and is a big leap towards all-magnetic computation.

## Methods

Magnetic vortex dynamics was simulated using finite difference method based Landau-Lifshitz-Gilbert (LLG) ordinary differential equation solver called Object Oriented Micromagnetic Framework (OOMMF). At first a magnetic ground state was obtained with the required polarity and chirality. This was accomplished by using a Gaussian pulsed field H_t_ = H_0_exp(−t′^2^), where μ_0_H_0_ = 1 T and normalized time t′ = (t − t_0_)/(√2σ), t_0_ = 75 ps and σ is the standard deviation of the Gaussian pulse in time whose full width at half maxima is 30 ps. To control the core polarity we have applied a field H_z_ = ± H_t_/10, along z direction, close to the centre of the circular disk, where the sign of the field determines the core polarity. X and Y components of the applied Gaussian field to produce the desired chirality are given by the equations 1 and 2 below:


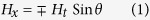



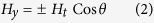


where θ = tan^−1^(y/x) and the upper or lower sign was chosen for the CCW and CW chirality, respectively. This pulse signal dies down quickly during the simulation. The simulation was run for 40 ns under a high damping (Gilbert damping constant α = 0.95 was used here) to obtain the ground state. The other material parameters used here are saturation magnetization *M*_*s*_ = 0.8 × 10^6^ A/m, exchange stiffness constant *A* = 13 × 10^−12^ J/m and zero magneto-crystalline anisotropy. During the vortex dynamics simulation we have used the value of damping as α = 0.008, which is a realistic value of damping for Py. The dynamic simulation was run for 40 ns and the average magnetization was observed in every 10 ps. The cell size used during simulation was 5 nm × 5 nm × 40 nm. We also performed simulations with cell size of 2 nm × 2 nm × 40 nm on the coupled two vortex and three vortex sequences and found that the results do not depend significantly on the cell size as long as the lateral dimensions of the cell size is below the exchange length of Py.

At first, the dynamics of single vortex was studied by using a broadband excitation signal and the natural frequency of the single vortex was found out. The excitation field is applied at t = 0. The signal had only X component *H*_*x*_^*S*^ which contained power up to *f*_*cut*_ = 45 GHz and dependent upon time as given by equation 3 as below:


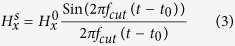


where, *μ*_*0*_*H*_*0*_ = 0.05 T and *t*_*0*_ = 200 ps.

For calculating the dynamics of two vortex and three vortex systems, we have applied an in plane rotating magnetic field of frequency 1.27 GHz at the left disk. After obtaining the data from OOMMF, we analyse the results by investigating the time evolution of spatial average of X component of magnetization, 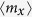
for each vortex, and its corresponding energy spectral density (ESD), 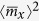. We normalize the magnetization by dividing the X component of magnetization *M*_*x*_ by *M*_*s*_. Subsequently, we performed Fourier transform to obtain the required ESD. This is plotted in decibel scale as 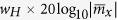, where a window scaling factor of *w*_*H*_ = 2 is used for the Hanning window. The Hanning window is used to minimize spectral leakage. These ESDs were calculated after running the dynamics for 40 ns so that any transient vortex core dynamics is suppressed and steady state dynamics becomes prominent in the spectrum. In various figures in this paper the ESDs are cut-off at arbitrary values to focus primarily upon the central mode with high value of ESD used for calculating the gain of transfer of energy. A lower cut-off of the ESD axis could show some additional modes with low ESD values but that does not affect the results presented in this paper. The stray field was also obtained from OOMMF during the dynamics. The stray field plots and the supplementary movies were created using MATLAB. The contour colour scale is logarithm of amplitude of in-plane components of the stray field which are normalized by 8 × 10^5 ^A/m. So the quantity being plotted is log_10_ (h_x_^2^ + h_y_^2^), where h_x_ = *H*_*x*_*/M*_*s*_ and h_y_ = *H*_*y*_*/M*_*s*_. Here *H*_*x*_ and *H*_*y*_ are the x and y components of the stray field and *M*_*s*_ (0.8 × 10^6 ^A/m) is the saturation magnetization of Py, used as normalization constant. The colour scale is in dB. The core shift of the vortex is found by fitting the out of plane component of magnetization profile with a Gaussian function and by finding the difference between the peak positions. The relative core shift as shown in Fig. 4(c) is the difference between the core shift of the input and output vortices. This relative core shift has possible errors originating from various sources. Consideration of finite cell size may introduce an error in the value of core shift. If the cell size is *d,* then the core position can be determined with a spatial resolution of ± *d*/2. The cell size determines the number of data points in the curve where z component of magnetization varies with distance giving the core profile. Another source of error comes from fitting of the core profile with Gaussian function itself. In addition to this, error can be introduced due to the limitation in the time step used in the simulation. The cell size and the precise determination of the core position may also affect the ESD. However, the primary source of error in ESD is the total time window used in the simulation, which determines the spectral resolution of discrete Fourier transform, used to obtain the ESD. Besides those, spectral leakage, despite the use of window function during the discrete Fourier transform, may affect the ESD.

## Additional Information

**How to cite this article**: Barman, S. *et al*. Enhanced Amplification and Fan-Out Operation in an All-Magnetic Transistor. *Sci. Rep.* **6**, 33360; doi: 10.1038/srep33360 (2016).

## Supplementary Material

Supplementary Movie 1

Supplementary Movie 2

Supplementary Movie 3

Supplementary Movie 4

Supplementary Movie 5

Supplementary Movie 6

Supplementary Movie 7

Supplementary Movie 8

Supplementary Movie 9

Supplementary Movie 10

Supplementary Movie 11

Supplementary Movie 12

Supplementary Movie 13

Supplementary Information
