# Supplementary material for: Enhanced Amplification and Fan-Out Operation in an All-Magnetic Transistor
Source: Sci Rep. 2016 Sep 14;6:33360. doi: 10.1038/srep33360 (PMC5022038; doi:10.1038/srep33360)
Supplement: Supplementary Information [file srep33360-s14.doc]

**Enhanced Amplification and Fan-Out Operation in an All-Magnetic Transistor**

Saswati Barman^1^, Susmita Saha^1^, Sucheta Mondal^1^, Dheeraj Kumar^1^ and Anjan Barman^1,*^
^1^Department of Condensed Matter Physics and Material Sciences, S. N. Bose National Centre for Basic Sciences, Block JD, Sec. III, Salt Lake, Kolkata 700106, India

^*^[abarman@bose.res.in](mailto:abarman@bose.res.in)

**Supplementary Figure**

**Supplementary Figure 1S: (a) ESDs for coupled vortex system with separation *S* = 10 nm and 60 nm for core polarities (+1, +1), (b) ESDs for AMVT (*S1* = 10 nm, *S2* = 100 nm) with different core polarization as shown in the top of the figure.**

We have performed test simulations on two vortex systems by changing the core polarities with separations of *S* = 10 nm and 60 nm. Figure 1S (a) shows that for polarities (+1, +1) the gain in the second vortex is lower than the one (+1, -1) presented in our manuscript. The corresponding gains are, 22 dB for *S* = 10 nm and 7 dB for *S* = 60 nm as opposed to 42.15 dB and 22.77 dB for (+1, -1).

For the three vortex system, we have performed test simulations for the AMVT with optimized separation (*S1* = 10 nm, *S2* = 100 nm) but different combinations of core polarities. Figure 1S (b) clearly shows that the combination of polarities (+1,-1,-1) used in this manuscript gives the maximum gain value in the output as opposed to the other combinations. The gain in output vortex is -4.5 dB, 5.0 dB and 16.5 dB for the polarity combination (+1, +1, +1), (+1, +1, -1) and (+1, -1, +1), respectively. Hence, we have chosen the polarities for the two vortex and AMVT (three vortex) as presented in the manuscript.

**Supplementary Movies**

| **Movie** | **File Name** | **Description** |
| --- | --- | --- |
| **M1** | movie1_2vortex_S=10nm | Time evolution of dynamic stray field for two vortex system with *S* = 10 nm. |
| **M2** | movie2_2vortex_S=30nm | Time evolution of dynamic stray field for two vortex system with *S* = 30 nm. |
| **M3** | movie3_2vortex_S=60nm | Time evolution of dynamic stray field for two vortex system with *S* = 60 nm. |
| **M4** | movie4_2vortex_S=100nm | Time evolution of dynamic stray field for two vortex system with *S* = 100 nm |
| **M5** | movie5_3vortex_S1=10_S2=10 | Time evolution of dynamic stray field for three vortex system with *S1* = 10 nm and *S2* = 10 nm. |
| **M6** | movie6_3vortex_S1=10_S2=50 | Time evolution of dynamic stray field for three vortex system with *S1* = 10 nm and *S2* = 50 nm. |
| **M7** | movie7_3vortex_S1=10_S2=80 | Time evolution of dynamic stray field for three vortex system with *S1* = 10 nm and *S2* = 80 nm. |
| **M8** | movie8_3vortex_S1=10_S2=100 | Time evolution of dynamic stray field for three vortex system with *S1* = 10 nm and *S2* = 100 nm. |
| **M9** | movie9_3vortex_S1=10_S2=150 | Time evolution of dynamic stray field for three vortex system with *S1* = 10 nm and *S2* = 150 nm. |
| **M10** | movie10_3vortex_S1=10_S2=175 | Time evolution of dynamic stray field for three vortex system with *S1* = 10 nm and *S2* = 175 nm. |
| **M11** | movie11_fan-out_Sx1=50_Sx2=50 | Time evolution of dynamic stray field for fan- out operation with *Sx1* = 50 nm and *Sx2* =50 nm. |
| **M12** | movie12_fan-out_Sx1=50_Sx2=30 | Time evolution of dynamic stray field for fan- out operation with *Sx1* = 50 nm and *Sx2* =30 nm. |
| **M13** | movie13_fan-out_Sx1=50_Sx2=10 | Time evolution of dynamic stray field for fan- out operation with *Sx1* = 50 nm and *Sx2* = 10 nm. |
